# Enforced effect of tk-MCP-1 fusion gene in ovarian cancer

**DOI:** 10.1186/1756-9966-31-74

**Published:** 2012-09-12

**Authors:** Shuhui Hong, Ping Zhang, Hui Zhang, Lin Jia, Xun Qu, Qifeng Yang, Fengnian Rong, Beihua Kong

**Affiliations:** 1 Department of Obstetrics and Gynecology, Qilu Hospital, Shandong University, 107 Wenhuaxi Road, Ji’nan, 250012, People’s Republic of China; 2 Department of Obstetrics and Gynecology, Affiliated Qianfoshan Hospital of Shandong University, 16766 Jingshi Road, Ji’nan, 250014, People’s Republic of China; 3 Department of Obstetrics and Gynecology, The Second Hospital of Shandong University, 247 Beiyuan Street, Ji’nan, 250033, People’s Republic of China; 4 Department of Basic Medicine Science, Qilu Hospital, Shandong University, 107 Wenhuaxi Road, Ji’nan, 250012, People’s Republic of China; 5 Department of Breast Surgery, Qilu Hospital, Shandong University, 107 Wenhuaxi Road, Ji’nan, 250012, People’s Republic of China

**Keywords:** Herpes simplex virus thymidine kinase, Monocyte chemoattractant protein-1, Gene therapy, Ovarian neoplasma, SCID

## Abstract

**Objective:**

The efficiency of HSV-tk/GCV system is not high because of insufficient gene transfer and incompletely initiative of host antineoplastic potency. The present study was designed to assess the antitumor efficacy of tk-MCP-1 on ovarian cancer in vitro and vivo.

**Methods:**

A novel bicistronic expression system can help to improve the expression level of a gene in a stable manner. pLXSN/tk-MCP-1 co-expressing tk and MCP-1 genes was constructed using a pLXSN retroviral vector and an internal ribosome entry site sequence by restriction enzyme. Western blot was performed to determine tk and MCP-1 expression in the infected SKOV_3_. The GCV-sensitively tumoricidal activities of SKOV_3_/tk-MCP-1 with or without monocytes were compared to those of SKOV_3_ expressing HSV-tk or MCP-1. We investigated the growth of subcutaneous tumors in SCID mice immuno-reconstituted, and evaluated the antitumor effect of MCP-1 in conjunction with suicide gene.

**Results:**

The significant GCV-sensitively tumoricidal activity of pLXSN/tk-MCP-1 was observed when compared with those of pLXSN/tk, pLXSN/MCP-1 and pLXSN/neo, especially when monocytes were added. The growth of subcutaneous tumors in SCID mice immuno-reconstituted was markedly suppressed by co-delivery of HSV-tk and MCP-1 genes, and the enhanced antitumor effect was associated with the recruitment of monocytes.

**Conclusion:**

These results demonstrated pLXSN/tk-MCP-1 presented an enhanced antitumor effects on ovarian cancer by orchestration of immune responses.

## Background

Ovarian cancer is the leading cause of death from gynecologic cancers. Every year, approximately 200,000 women are diagnosed with ovarian cancer and more than 100,000 women died of ovarian cancer around the world [1,2]. Due to inherent and acquired chemoresistance of ovarian cancer, the effect of current therapies for advanced or metastatic ovarian cancer is far from satisfying [3]. This underscores the imperative to adopt new strategies to fight against ovarian cancer effectively.

Suicide gene therapy is one of these strategies with antitumor effect [4,5]. However, its efficacy for the treatment of cancer is limited because of the insufficient gene transfection and insufficient induction of host immunity [6-8] .

The bystander killing effect is a mechanism counting on host immunological function, which could kill the neighboring uninfected tumor cells produced by suicide gene HSV-tk/GCV system and finally strongly enhance the capacity against the tumor cells [9,10]. Recently, increasing studies have been carried out to optimize the suicide gene therapy in combination with immune genes.

MCP-1 is one of thte chemokine responsible for the recruitment and activation of mononuclear cells, and it can induce nonspecific and specific antitumor immunity [11,12]. Therefore, we hypothesized that tk-MCP-1 fusion gene could significantly enhance the efficacy of suicide gene therapy contributed by the direct antitumor activity and the elicited anti-tumor immunity in ovarian cancer.

## Materials and methods

### Recombinant retroviruses

**Figure 1:**
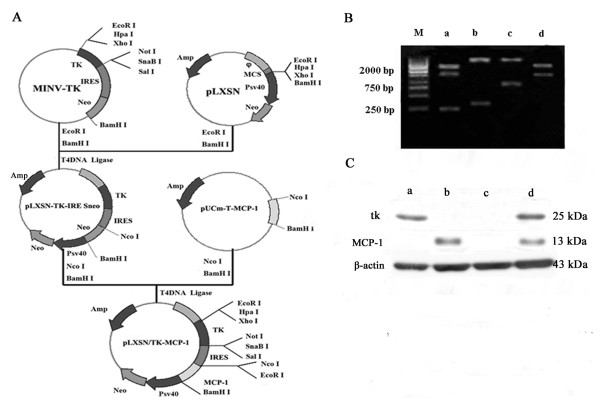
**The plasmid characterization and confirmation of expression of tk and MCP-1 by RT-PCR and western blot. A**. The construction of the bicistronic recombinant replication-defective retroviruses vector pLXSN/tk-MCP-1, pLXSN/tk and pLXSN/MCP-1. **B**. Restriction enzyme analysis of pLXSN/tk-MCP-1 showed that tk and/or MCP-1 gene fragment had insert in the proper orientation in the vector of pLXSN, pLXSN/tk, pLXSN/MCP-1 and pLXSN/neo. a: EcoR I + BamH I, b: Nco I + BamH I, c: Sal I + BamH I, d: EcoR I, M: Marker. **C**. The expression of tk and MCP-1 protein were detected by western blot 48 h after transfection. a: SKOV_3_/tk. b: SKOV_3_/MCP-1. c: SKOV_3_/neo. d: SKOV_3_/tk-MCP-1.

We designed the PCR or RT-PCR primers for HSV-tk, MCP-1 and IRES. HSV-tk: 5^′^-GCGCGTATGGCTTCGTACCC-3^′^ and 5^′^-TCCTTGCGTGTTTCAGTTAGTC-3^′^. MCP-1: 5^′^-CGGAATTCATATGCAGCCAGATGCAATC-3^′^ and 5^′^-CGGGATCCTTA TCAAGTCTTCGGAGT-3^′^. IRES: 5^′^- CGATCGATCTCCACGTGGCGGC-3^′^ and 5^′^- CCTGATAATCCAATTCGCTTTAT-3^′^. Total RNA was extracted from human peripheral blood mononuclear cells (PBMC) followed by RT-PCR to generate MCP-1 gene fragment with 5 min at 95°C, 1 min at 94°C, 1 min at 58°C and 1 min at 72°C, up to 35 cycles. By Restriction Enzyme cutting site, EcoRI - XhoI internal ribozyme entry site (IRES) fragment of poliomyelitis virus, we got linear pLXSN. Then it was inserted into the herpes simplex virus thymidine kinase gene fragment from pWZLneotkglyCD with BamHI-EcoRI to generate the tk-IRES-neo, and pLXSN/tk was obtained by insertion of tk-IRES-neo into Linear pLXSN. pLXSN fragment combined with MCP-1 gene fragment to generate pLXSN/MCP-1. MCP-1 gene fragment was inserted into pLXSN/tk-IRES-neo to form pLXSN/tk-MCP-1. The above plasmids were verified by PCR. Retroviruses containing pLXSN/tk-MCP-1, pLXSN/tk, pLXSN/MCP-1 and pLXSN/neo respectively were generated by transfecting PA317 cells using liposome, and transfected cells were selected by G418 at diverse concentrations. The titer of retrovirus was determined (Figure 1-A).

### RT-PCR

Total RNA was extracted as described previously and RT-PCR was performed comprising 33 thermal cycles of 95°C for 5 min, 94°C for 1 min, 58°C for 1 min, 72°C for 1 min and 72°C for 7 min. The same condition was used in MCP-1 amplification except 30 cycles in total.

### Cell culture and retrovirus infection

The human epithelial ovarian cancer cell line SKOV_3_ was used in vitro and vivo. SKOV_3_ cells were infected with supernatant of retrovirus at high titre containing pLXSN/tk-MCP-1(5.3 × 10^5^ CFU/ml), pLXSN/tk(6.0 × 10^5^ CFU/ml), pLXSN/MCP-1(4.8 × 10^5^ CFU/ml) and pLXSN/neo(4.5 × 10^5^ CFU/ml) at various volumes (100 μl, 200 μl, 500 μl or 1 ml), supplied with RPMI-1640 with 10% NBS to 2 ml, and then added polybrene (the concentration of polybrene at 8 μg/ml). Three hours later, cells were supplied with RPMI-1640 with 10% NBS to 8 ml and cultured for 2–3 days at 37°C in a 5% CO_2_ atmosphere. G418 at 600 μg/ml was added into 4 kinds of cells. Ten days later, cells which survived in medium containing G418 at 600 μg/ml named SKOV_3_/tk-MCP-1, SKOV_3_/tk, SKOV_3_/MCP-1 and SKOV_3_/neo.

### Western blot

Proteins were extracted using protein extraction reagent, 48 h after transfection and save at −20°C, following a protocol provided by the manufacture. MCP-1 protein and tk protein expressions were detected with western blot. Proteins with equal amount were separated by appropriate concentration SDS-polyacrylamide gel electrophoresis and transferred onto PVDF membrane (Millipore, Billeriaca, MA, USA). The membranes were blocked in TBST for 1 h at room temperature and then incubated with primary antibodies fo tk (1:500, Abcam, United Kingdom), MCP-1 (1:500, Santa Cruz Biotechnology) and β-actin (1:5000, Boston, MA) overnight at 4°C The membranes were then washed three times with TBST, followed by incubating with HRP-labeled secondary antibodies (KPL, Gaithersburg, MD, USA) (1:5000). Bound antibody was visualized using ECL detection reagent (Merck, Darmstadt, Germany).

### Antitumor effect of GCV

The number of viable cells were determined by 3-(4, 5-dimethylthiazol-2-yl) -2,5-diphenyl tetrazolium bromide (MTT) assay. There were 4 experimental groups including SKOV_3_/tk, SKOV_3_/MCP-1, SKOV_3_/tk-MCP-1 and SKOV_3_/neo. Cells were re-suspended in fresh culture medium at the density of 2 × 10^4^ cells/ml, 180 μl suspension were incubated in 96-well plates. The cells were treated with 20μl GCV at the concentrations of 10^−2^, 10^−1^, 1, 10, 10^2^, 10^3^ μg/ml for 72 h at 37°C in 5% CO_2_ incubator. SKOV_3_/tk-MCP-1 and SKOV_3_/neo seeded by same way was added GCV (1.0 μg/ml, 0.1 μg/ml) incubated for 24, 48, 72 and 96 h to detect time toxicity of GCV. 20μl Sodium Chloride was added to controls. The activity of the mitochondria, reflecting cellular growth and viability, was evaluated by measuring the optical density at 490 nm on microtiter plate reader.

We also detected the antitumor effect of human monocytes on gene modified ovarian cells by MTT: There were 3 experimental groups including SKOV_3_/MCP-1, SKOV_3_/tk-MCP-1 and SKOV_3_/neo. Mononuclear cells were used as effectors, and tumor cells above-mentioned were used as target. Cells were seeded in the 96-well plates at the density of 5 × 10^3^ cells/well. Then mononuclear were added at different ratio of effector to target (20:1, 10:1, 5:1), incubated at 37°C in 5% CO_2_ incubator for 4 days, cytotoxicity were determined.

The surviving rate of mixed tumor cell under the action of GCV only was determined by MTT. Briefly, there were 3 experimental groups (including SKOV_3_/tk, SKOV_3_/tk-MCP-1 and SKOV_3_/neo). The above cells infected by different gene at different proportion (100%, 90%, 70%, 50%, 30%, 10%, 0) were mixed with wild SKOV_3_, and then were added in 10 μg/ml GCV The surviving rate of cells were determined by MTT incubated in 96-well plates for 4 days at 37°C in 5% CO_2_ incubator.

Next we detect the surviving rate of mixed tumor cell under the action of GCV plus human monocytes by MTT. Each kind of cells and wild SKOV_3_ were seeded in 96-well plates as the same way. Then 5 × 10^4^ human monocytes were added at the ratios of 10:1(effectors: target). All cells were incubated for 4 days at 37°C in 5% CO_2_ incubator after supplied 10 μg/ml GCV. Cells without GCV were used as control group.

Detection of cell apoptosis rate, cell cycle and the expression of CD25 (IL-2R) and CD44v6 by flow cytometer: SKOV_3_/tk, SKOV_3_/tk-MCP-1 and SKOV_3_/neo were seeded in 25 cm flask. After cells adherenced, we added human monocytes at the ratios of 10:1(effectors: target) and 0.5 μg/ml GCV, and then incubated cells for 48 h at 37°C in 5% CO_2_ incubator.

### Animal experiments

The present study was approved by the local animal Care Committee and is in compliance with Chinese laws for animal protection. 6 to 8 weeks old, weight-matched female combined immune deficiency mice (C.B17/SCID) were purchased from Weitonglihua experimental animal limited company. Animals were housed in the animal facility of the Medical College of Shandong university of China. Enzyme-linked immunosorbent assay (ELISA) for the IgG of C.B17/SCIDs in serum was performed to eliminate immune leakage according to the manufacturer’s protocol. Human mononuclear cells were isolated from human peripheral blood mononuclear cells by Ficoll-Hypaque discontiguous density gradient centrifugation technique and were re-suspended in fresh RPMI 1640 medium without NBS at a density of 8 × 10^7^cells/ml. 0.5 ml cell suspension was injected into abdominal cavity of per C.B17/SCID for immunologic reconstitution. Twenty-four hours after celiac immunologic reconstitution, SKOV_3_/neo, SKOV_3_/tk, SKOV_3_/MCP-1 and SKOV_3_/tk-MCP-1 cell lines were inoculated by intraperitoneal injection at a density of 2 × 10^7^ cell/SCID. According to the cells inoculated, all experimental C.B17/SCIDs were divided into 4 groups, i.e. SKOV_3_/neo, SKOV_3_/tk, SKOV_3_/MCP-1 and SKOV_3_/tk-MCP-1 group (10 SCIDs/group). SKOV_3_/neo group was used as control group and the rest groups were experimental groups. We injected GCV 75 mg/kg·d intraperitoneally for 5 days after tumor transplantation, then, observed the biologic characteristics of SCID, such as spirit, appetite and abdominal bulge.

The survival periods of 4 SCID mice selected randomly from each groups were recorded from being successfully transplanted human ovarian carcinoma cells to natural death. The rest 6 SCID mice of each groups were sacrificed as soon as the appearance of death in the control group.

The number of macrophages infiltrated the tumor sites was examined by flow cytometry. Briefly, monoplast suspension of tumor tissue was prepared by trituration. Cells were re-suspended in PBS at the density of 1 × 10^6^ cells/ml followed by addition of 10 μl human CD14/PE (Pharmingen USA) antibody mixing thoroughly. After 30 min of activation away from light at 20°C-25°C, flow cytometry was used to detect the amount of macrophages. The TNF-α protein level was analysised by western blot.

The cell apoptosis rate, cell cycle and the expression of CD25 (IL-2R) and CD44v6 in tumor cells were detected by flow cytometer.

### Statistical analysis

The SPSS version 13.0 software was used for statistical analysis. Results were reported as means ± standard deviation (SD). The statistical differences between group was assessed by q test. Kaplan-Meier survival curves were generated with the use of SPSS 13.0. Comparisons of median survivals were performed using log-rank tests. Alpha (α) level was set at 0.05.

## Results

### Confirmation of plasmid

Restriction enzyme analysis of plasmid DNA showed that tk and MCP-1 gene fragment were inserted in the proper orientation in the vector of pLXSN named pLXSN/tk-MCP-1, so had pLXSN/tk, pLXSN/MCP-1 and pLXSN/neo (Figure 1-B).

### Packaging and transfection of pLXSN/tk, pLXSN/MCP-1, pLXSN/tk-MCP-1 and pLXSN recombinantretroviral vector

The recombinant retroviral vectors including pLXSN/tk, pLXSN/MCP-1, pLXSN/tk-MCP-1 and pLXSN/neo, were transfected into retroviral packaging cell line PA317 by DOTAP, respectively. Stable retroviral vector-produced lines were generated by expanding the G418-resistant (> 500 μg/ml) colonies, named PA317/tk (pLXSN/tk transferred), PA317/MCP-1 (pLXSN/MCP-1 transferred), PA317/tk-MCP-1(pLXSN/tk- MCP-1 transferred) and PA317/neo (pLXSN transferred) respectively. The supernatant containing the packaged retroviruses was harvested, filtered and titrated 4.5 × 10^5^ CFU/ml-6.0 × 10^5^ CFU/ml determined in NIH3T3 cells. SKOV_3_ cells were infected with the high titre recombinant retrovirus (pLXSN/tk, pLXSN/MCP-1, pLXSN/tk-MCP-1 and pLXSN/neo), while SKOV_3_ tansfected pLXSN/neo was used as the control group. Stable retroviral vector-produced cell lines were generated by expanding the G418-resistant (600 μg/ml) colonies, named SKOV_3_/neo, SKOV_3_/tk, SKOV_3_/MCP-1 and SKOV_3_/tk-MCP-1 respectively.

### Validation of tk and MCP-1 expression

Western blot analysis demonstrated that MCP-1 protein was expressed in the SKOV_3_/MCP-1 and SKOV_3_/tk-MCP-1, and tk protein was expressioned in the SKOV_3_/tk and SKOV_3_/tk-MCP-1 (Figure 1-C).

### Antitumor effect

**Figure 2:**
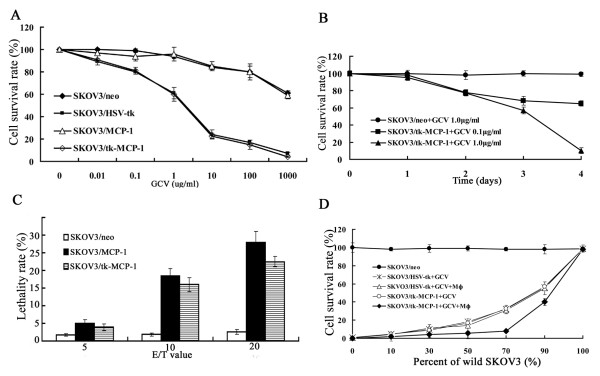
**Antitumor effection. A**: MTT assay of GCV on ovarian cancer cells. **B**: GCV at the density of 0.1 μg/ml, the beginning cytotoxic was 48 h and 40% kill rate at 96 h, however, the beginning cytotoxic was 48 h and 90% kill rate at 96 h when GCV at the density of 1.0 μg/ml. **C**: Lethal effect of mononuclear macrophage on SKOV_3_/MCP-1 and SKOV_3_/tk-MCP-1 was determined by MTT assay. **D**: There is a synergistic antitumor effect when cooperated tk-MCP-1 + GCV system with mononuclear macrophage.

As shown in Figure 2-A, the viability of cells dose-dependently reduced. GCV at the density of 10^-2^-10^3^ μg/ml had obvious antitumor effect on SKOV_3_/tk (IC50:2.24 ± 0.23 μg/ml) and SKOV_3_/tk-MCP-1 (IC50:2.06 ± 0.31 μg/ml). The IC50 value of SKOV_3_/tk and SKOV_3_/tk-MCP-1 significantly dropped when compared to that of SKOV_3_/neo (*P* < 0.05). There was no significant difference between SKOV_3_/MCP-1 group and control groups (*P* > 0.05). Besides, the beginning cytotoxic time of 0.1 μg/ml GCV and 1.0 μg/ml GCV was both 48 h, and the 96 h kill rate of 0.1 μg/ml GCV and 1.0 μg/ml GCV against SKOV_3_/tk-MCP-1 was 40 ± 2.19% and 90 ± 4.55% respectively (*P* < 0.05) (Figure 2-B).

The antitumor effect of monocytes on ovarian cancer cells: The maximum lethality rate of SKOV_3_/MCP-1 and SKOV_3_/tk-MCP-1 was 29 ± 1.25% and 23 ± 2.18% respectively, comparing to 1.8 ± 0.64% of SKOV_3_/neo (P < 0.05). We found that the lethal effect of monocytes on tumor cells was effector-dependent, and the maximum lethality rate appeared at the ratio of 20:1(Figure 2-C).

The survival rate of SKOV_3_/tk and SKOV_3_/tk-MCP-1 incubating with SKOV_3_ in different ratio was evaluated after addition GCV or GCV plus monocytes (Figure 2-D). When 10 μg/ml GCV was added, only 10% of SKOV_3_/tk or SKOV_3_/tk-MCP-1 could kill about 40% of tumor cells. When the ratio of SKOV_3_/tk or SKOV_3_/tk-MCP-1 to SKOV_3_ was 50%, there were about 80% of tumor cells killed. But cytotoxin did not appear with SKOV_3_/neo(*P* < 0.05). Only 10% of tk-MCP-1 + GCV + monocytes system could kill about 70% of tumor cells, while 40% of tk-MCP-1 + GCV + monocytes system could kill about 90% of tumor cells.

The result of flow cytometer showed that the apoptotic rate of SKOV_3_/tk-MCP-1 (13.48 ± 1.01%) was obviously higher than those of SKOV_3_/tk (9.50 ± 1.33%) and SKOV_3_/neo (2.19 ± 0.56%) (*P* < 0.05), S phase of SKOV_3_/tk (38.31 ± 1.67%) was lower than that of SKOV_3_/tk-MCP-1 (52.92 ± 1.78%) (*P* < 0.05)(Table 1).

**Table 1:** Post-treatment apoptotic rate and cell cycle analysis ($\bar{\text{x}}\pm \text{s}$**)**

	**SKOV**_3_**/neo**	**SKOV**_3_**/tk**	**SKOV**_3_**/tk-MCP-1**
Apoptotic rate (%)	2.19 ± 0.56	9.50 ± 1.33	13.48 ± 1.01
G0/G1 (%)	53.90 ± 1.66	53.10 ± 1.21	40.28 ± 1.11
S (%)	19.34 ± 0.65	38.31 ± 1.67	52.92 ± 1.78
G_2_/M (%)	26.76 ± 1.01	8.59 ± 1.25	6.80 ± 1.11

**Figure 3:**
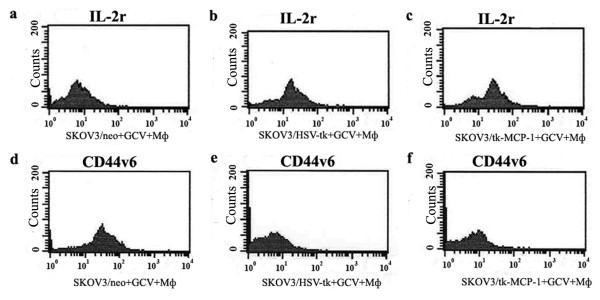
**CD**_**25**_**of SKOV**_**3**_**/tk (20.00 ± 2.04%) and SKOV**_**3**_**/tk-MCP-1 (38.82 ± 2.48%) was obviously higher than SKOV**_**3**_**/neo (8.73 ± 1.65%) (*P*** **< 0.05).** CD_25_ of SKOV_3_/tk-MCP-1 was significantly higher than that of SKOV_3_/tk (*P* < 0.05). CD_44v6_ of SKOV_3_/tk (6.66 ± 2.01%) and SKOV_3_/tk-MCP-1 (6.51 ± 1.03%) was significantly lower than that of control group (40.74 ± 3.58%) (*P* < 0.01)

The percentage of positive cell represents content of CD25 (IL-2r) or CD44v6 (Figure 3), CD25 of SKOV_3_/tk (20.00 ± 2.04%) and SKOV_3_/tk-MCP-1 (38.82 ± 2.48%) was obviously higher than that of SKOV_3_/neo (8.73 ± 1.65%)(*P* < 0.05). CD25 of SKOV_3_/tk-MCP-1 was significantly higher than that of SKOV_3_/tk (*P* < 0.05). CD44v6 of SKOV_3_/tk (6.66 ± 2.01%) and SKOV_3_/tk-MCP-1 (6.51 ± 1.03%) was significant lower than that of control group (40.74 ± 3.58%) (*P* < 0.01).

### Antitumor effects of recombinant gene in vivo

**Figure 4:**
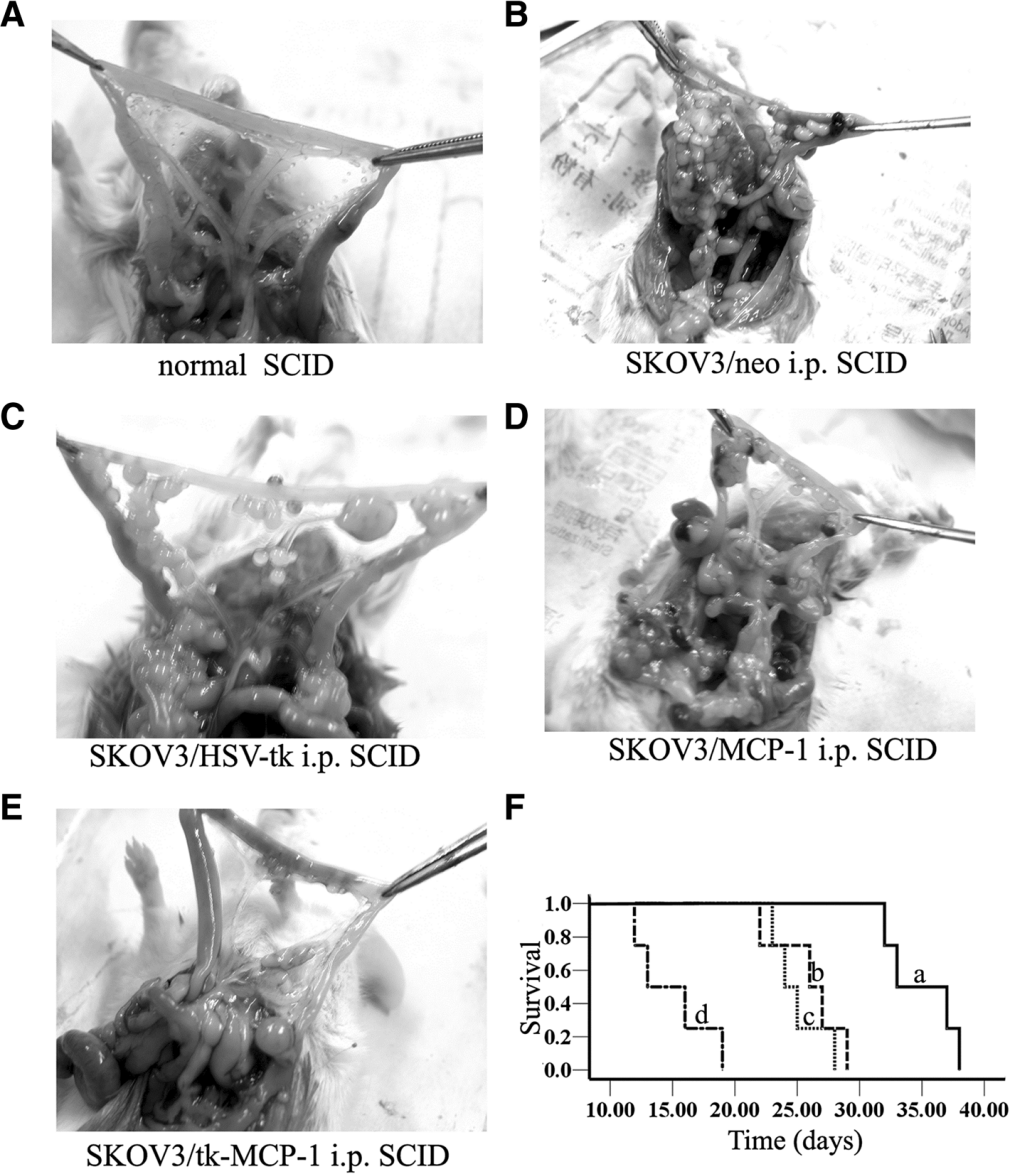
**A–E. The ovarian tumors of the tk-MCP-1 group shrank significantly, followed by the tk or MCP-1 group.** However, the tumor of the control group was still widespread in peritoneal cavity and cavitas pelvis. **F**. Kaplan-Meier survival analysis of mice intraperitoneally transplanted with diverse tumor cells. a. SKOV_3_/tk-MCP-1 b. SKOV_3_/MCP-1 c. SKOV_3_/tk d. SKOV_3_/neo.

**Figure 5:**
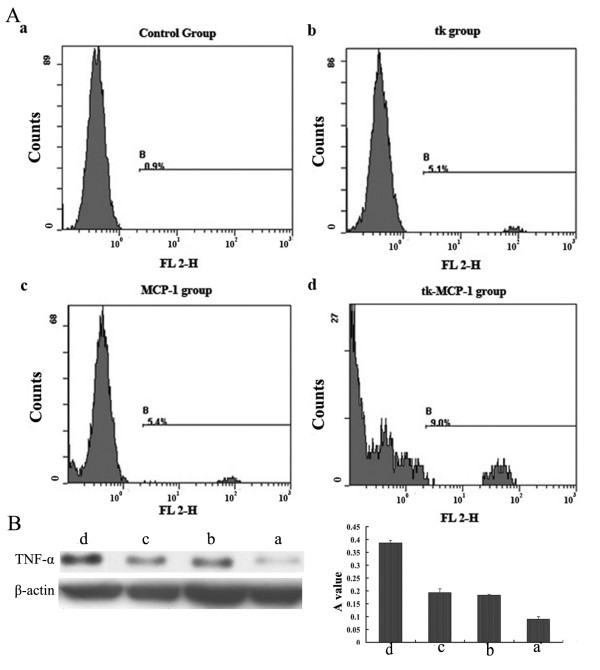
**Flow cytometry examination revealed that the number of macrophages (A) infiltrated the tumor tissues in the control group, tk or MCP-1 group and tk-MCP-1 group increased in order (*P*<0.05), so did TNF-α protein level from the activated microphages.** There was no significant difference between tk and MCP-1 groups (*P* > 0.05) (**B**). a. SKOV_3_/neo b. SKOV_3_/tk c. SKOV_3_/MCP-1 d. SKOV_3_/tk-MCP-1.

Forty SCIDs (IgG < 5 μg/ml) were injected PBMC intraperitoneally. Three weeks later immune reconstruction was successfully established in SCID mouse (human IgG > 5 μg/ml). The ratio of successful tumor transplantation was 100%. The tumor was widespread in peritoneal cavity. The reduction of abdominal bulge and the improvement of spirit and appetite of tk-MCP-1group were greater than tk or MCP-1, and the condition of control group had no amelioration. The survival period of tk-MCP-1 group was significantly longer than tk or MCP-1 group, followed by the control group (35 ± 2.94 d, 25 ± 2.16 d, 26 ± 2.58 d and 15 ± 3.16 d, *P* < 0.05). There was no significant difference between tk and MCP-1 groups (*P* > 0.05) (Figure 4-F). The ovarian tumors of the tk-MCP-1 group shrank significantly, followed by the tk or MCP-1 group. However, the tumor of the control group was still widespread in peritoneal cavity and cavitas pelvis There was no significant difference between tk and MCP-1 groups (*P* > 0.05) (Figure 4A–E). As shown in Figure 5 and Table 2, flow cytometry examination revealed that the number of macrophages infiltrated the tumor tissues in the control group, tk or MCP-1 group and tk-MCP-1 group increased in order (*P* < 0.05), so did TNF-α protein level from the activated microphages. There was no significant difference between tk and MCP-1 groups (*P* > 0.05).

**Table 2:** Mean Percent of macrophages in each group

**Groups**	**Percent of macrophages (%) (**$\bar{\text{x}}\pm \text{s}$**)**
tk-MCP-1	8.70 ± 0.35
MCP-1	5.20 ± 0.28
HSV-tk	4.90 ± 0.24
The control group	0.90 ± 0.25

## Discussion

It is clear that expression of a single transgene is unlikely to be sufficient to eradicate ovarian cancer that is diagnosed late in disease progression. Many studies have demonstrated that HSV-tk combined with cytokine therapy followed by GCV has a higher chance of success [13-18]. MCP-1 (CCL2) has been successfully used to treat hepatocellular carcinoma by recombinant adenovirus vector (rAd)s expressing with HSV-tk [19]. Because several preclinical studies have demonstrated that genotoxic potential is not identical among all retroviral vector systems [20], and IRES could enable two different gene expressed simultaneously [21], we constructed pLXSN/tk-MCP-1 which co-expresses tk and MCP-1, and assessed the antitumor effect of pLXSN/tk-MCP-1 on ovarian cancer.

MCP-1 plays a crucial role in tumor tissue inflammatory response by activating and inducing the infiltration of macrophages, and in the regulation of adhesion factors expression which causes the contact ot macrophages with tumor cells. Once the effector cells get close to target cells, macrophages present the effect of antitumor by swallowing and killing pathogen, corpus alienum, senile and mutant cells, participating in nonspecific immune reaction and specific immunity, dealing with antigenic properties and presenting antigenic information to T or B lymphocyte [22-24]. Yamashiro et al. [25] found that the increasing amount of activated peripheral blood monouclear cells transfected MCP-1 gene infiltrating in tumor could restrain the growth of tumor. The present study suggested that MCP-1 could activate human mononuclear macrophage and carries a role in antitumor reaction, but the growth of tumor cells in control group was scarcely refrained. The more the effector cells, the stronger the tumoricidal effect of mononuclear macrophage was. Here our data provided strong evidence that MCP-1 had the antitumor reaction by activating mononuclear macrophage.

Bystander effect plays an important role in suicide gene therapy of tumor. Many studies have demonstrated that bystander effect might be due to immunization. Ramesh et al. [26] confirmed that the integrity of host immune was essential for suicide gene therapy. They performed RT-PCR after HSV-tk + GCV treatment and found the release of cytokines (TNF-α, IL-1, IL-6, IFN-α and GM-CSF mRNA) consistently increased [27].

Immunohistochemical analysis for tumor tissue after HSV-tk/GCV treatment showed a great quantity of CD4^+^, CD8^+^ lympholeukocyte recruiment. Gagandeep et al. [28] found that many immunocells infiltrated in tumor after HSV-tk + GCV therapy and cytokines released to cause hemorrhagic necrosis of tumor. The externalization of these cytokines depended on tumor cytotoxic effect and revoked up-regulation of immunological regulators such as MHC, B7 and ICAM-1. Accordingly, the mechanism of bystander effect depends on the transition of tumor microenvironment from immune suppression to immune activated state which initiates anti-tumor effect of immunal inflammatory system.

The use of cytokine gene therapy combined with suicide gene/prodrug can enhance bystander effect by reinforcing immune function. Chen et al. [29] injected recombined adenovirus expressed both IL-2 gene and HSV-tk gene to colon carcinoma model with hepatic metastasis, and found that the link of tk gene and IL-2 possess was more sufficient than individual gene therapy.

We demonstrated that the therapy of a combining suicide gene (HSV-tk and MCP-1) significantly improved the antitumor efficiency on SKOV_3_ cells by bicistronic recombinant replication-defective retroviruses vector pLXSN/tk-MCP-1 constructed in our lab. The bicistronic pLXSN co-expressing tk and MCP-1 linked by a bicistronic unit including poliomyelitis virus IRES was designed by proteinaceous translation initiation model inside chain of eukaryotic cell. The single upstream promoter can transcribe the same mRNA from two genes, and then the gene in the upstream is translated in eukaryocyte cap-dependent manner, while the downstream gene can be translated and expressed under the control of IRES in cap-independent manner, avoiding the influence on expression of the two genes at the regulation level of transcription.

The maximum concentration of MCP-1 and the result of chemotactic index of MCP-1-mediated migration showed that SKOV_3_/tk-MCP-1 could secrete MCP-1 possessed chemotactic activity. Furthermore, our study showed a strong bystander effect was observed in the system of SKOV_3_/tk-MCP-1 + GCV. MCP-1 is one of the major chemoattractants for mononuclear macrophage which can directly eradicate tumor cells, the importance is MCP-1 significantly induced a low survival rate when transduced cells and untransduced cells are cultured together in specific ratios as a immuno-modulator. Boosted bystander effect by immunal inflammatory system showed 10% tk + can induce a 70% tumor cells death rate. Combined HSV-tk with MCP-1 gene therapy is a powerful approach for the treatment of ovarian cancer. They not only could play their antitumor role respectively, but also could creat synergistic action which could enhance the anti-tumor immune reactions. Many immune effector cells aggregate to tumor site via the expression of MCP-1 activity and provoke nonspecific immune reaction and specific immunity, not only boosting the cytotoxic effect of GCV but also enhancing immune reaction to reinforce the bystander effect.

In order to explore the synergic antineoplastic mechanism and the influence on tumorous biological behavior of combined HSV-tk and MCP-1 gene, we investigated apoptosis and cell cycle. The results indicated that with the depressant effect of treatment on ovarian cells, FCM emerged manifest apoptotic peak, and that the proportion of S stage phase in cell cycle significantly increased at the same time compared with the control group. We also detected that the apoptosis rate of SKOV_3_ caused by HSV-tk-MCP-1 + GCV (13.48 ± 1.01%) was significant higher than that of HSV-tk + GCV (9.50 ± 1.33%). Similarly, the proportion of S stage of the former markedly increased than the latter.

These studies open the possibility that the prodrug GCV can blockage the cell cycle at S stage. The fact that the expression of CD25 significant raised after SKOV_3_ transfected tk-MCP-1 gene detected by FACS suggests that the immunogenicity of tumor cells may be enhanced after the treatment of combined tk and MCP-1 gene therapy. A study showed that the abnormal expression of adhesion molecule of cell surface CD44 and its var CD44v6 is closely related to infiltration, metastasis and dys-prognosis of malignancy [30,31]. We also demonstrated that the expression of CD44v6 was significantly lower after the administration of GCV on tumor cells successfully transfected SKOV_3_/tk and SKOV_3_/tk-MCP-1 gene, which suggests that suicide gene therapy may retroconverse the infiltration, metastasis of malignant cells and the expression of MCP-1 has no significant effect.

Freeman and colleagues [32] reported that suicide gene therapy could shift tumorous microenvironment from immune suppression to immunostimulation in order to initiate antitumor effect by inflammation, indicating that bystander effect relies in part on an intact immune system following tk/GCV gene therapy. We used SCID mouse as tumor vehicle, which had defect in both cellular and humoral immune function, to explore the antitumor mechanism of human immunal system. SCID mouse is an ideal preclinical empirical animal model because it can either load human tumor or be immunal functional reconstructed by human immunocyte. In this study, SKOV_3_/tk, SKOV_3_/MCP-1 or SKOV_3_/tk-MCP-1 cell line was intraperitoneally transplanted after immune reconstruction being successfully established in SCID mouse 3 weeks after intraperitoneally transplantation of PBMC. The tumor was widespread in peritoneal cavity, mainly in diaphragm, liver and mesentery. We demonstrated that tk-MCP-1 fusion gene had significantly tumoricidal effect in vivo partly depending on the effector of TNF-α from the activated of mononuclear macrophages induced by MCP-1.

## Conclusions

In conclusion, our data suggest that combined suicide gene therapy with immune gene therapy generates significantly stronger therapeutic antitumor effects by different mechanism and distinct link. This research provided sound evidence for preclinical research of ovarian carcinoma treatment, and might become the theoretical of a novel therapeutic strategy.

## Competing interests

The authors declare that they have no competing interests.

## Authors’ contributions

SHH, FNR and BHK made conception, designed and coordinated the study, carried out data interpretation, and drafted the manuscript; PZ and HZ participated in the conception and design of the study, and participated in drafting of manuscript; LJ participated in the design of the study and performed the statistical analysis; XQ and QFY conceived of the study, and participated in its design and coordination and helped to draft the manuscript. All authors read and approved the final manuscript.
